# Carbon Reporting Practices in the NHS: Emissions and Omissions Relating to Artificial Intelligence

**DOI:** 10.2196/79174

**Published:** 2025-10-27

**Authors:** Duncan J Reynolds

**Affiliations:** 1 Wolfson Institute of Population Health Queen Mary University of London London United Kingdom

**Keywords:** artificial intelligence, NHS, carbon reporting, health care, net zero

## Abstract

Artificial intelligence (AI) is being rolled out across the UK National Health Service (NHS) to improve efficiency; yet, its carbon footprint is largely invisible within mandatory Green Plan reporting. This work shows where NHS carbon reporting omits AI-related emissions and proposes feasible accounting and procurement measures that allow trusts to assess whether AI adoption advances or undermines net zero. A review of NHS sustainability guidance, the Department for Environment, Food & Rural Affairs conversion factors, and recent evidence on AI energy use shows that current Scopes 1-3 accounting omits substantial emissions at 3 points. First, a lack of granularity provides averages that can obscure the extreme energy intensity of certain AI workloads. Second, life-cycle emissions from specialized hardware (eg, graphics processing units) are often excluded unless trusts own the equipment, ignoring upstream manufacturing impacts. Third, widespread use of unprocured generative AI tools is unmeasured; extrapolating general practice survey data suggests that ChatGPT queries alone could release ≈ 349t CO₂e per year in primary care. To close these gaps, we propose three potential ways to help reduce these reporting gaps: (1) AI-specific carbon disclosure clauses in vendor contracts, (2) inclusion of cradle-to-grave emission factors for AI hardware in Scope 3 reporting, and (3) lightweight monitoring of external AI traffic (while recognizing potential ethical issues with this). Implementing these measures would give health care leaders a more accurate baseline against which to judge whether AI supports or undermines the NHS net-zero target.

## Why Artificial Intelligence Matters for NHS Net Zero

Artificial intelligence (AI) is increasingly integrated in health care delivery, including within the UK’s National Health Service (NHS) [1]. Although AI promises more efficient care [2] and potential climate benefits [3], it also carries a carbon cost due to the energy-intensive computation and manufacturing of digital infrastructure​ [4]. On the one hand, AI-driven automation could reduce emissions by streamlining care processes, optimizing resource use, and supporting remote care. On the other hand, AI systems (particularly large-scale models) consume significant energy and contribute to carbon emissions through data center operations, hardware production, and software training. Given that the NHS accounts for 4% of United Kingdom’s total carbon emissions [5], it is crucial to understand how AI aligns with its sustainability goals. This can only be done if the reporting of carbon emissions from AI is accurate. This work will focus on carbon emissions reporting in the NHS and how AI troubles current practice in order to show where current reporting omits AI-related emissions and proposes feasible accounting and procurement measures that allow trusts to better measure AI emissions.

**Figure 1:**
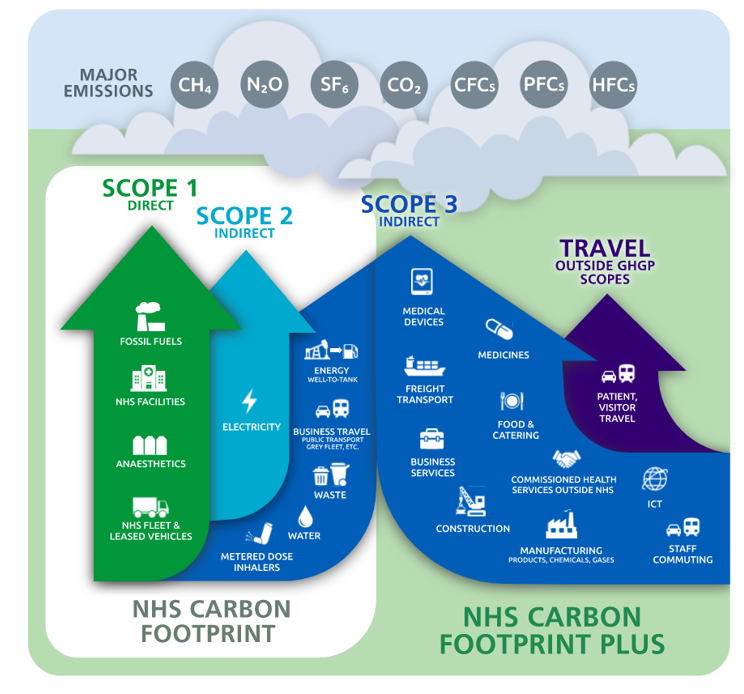
A visual of the greenhouse gas protocol scopes in the context of the NHS (reproduced from NHS England [5], which is published under open government license [8]).

The NHS has committed to achieving net zero emissions by 2040 for direct operations (Scopes 1 and 2) and by 2045 for indirect emissions (Scope 3), as part of the United Kingdom’s wider goals of net zero by 2050 [6]. A visual of these from NHS England can be seen in Figure 1 [5]. Current reporting frameworks identify emissions within these scopes, but critically, there is no specific accounting for the emissions associated with AI technologies, potentially leaving a significant gap in NHS’s net zero roadmap. In this piece, I argue that the current NHS’s net zero strategy potentially underestimates or overlooks the carbon footprint of AI. Without proper measurement and regulation (such as AI carbon accounting and sustainable procurement), rising AI adoption could undermine NHS’s environmental goals. The focus of this viewpoint is not whether AI will be environmentally positive or negative for the environment and the NHS (which has been covered elsewhere, such as [3,7]) but rather that current reporting practices are not currently stringent enough, given the increased use of AI in the NHS.

## NHS Net Zero Commitments and Reporting

The NHS has set ambitious net-zero targets, now embedded in law via the Health and Care Act 2022 [9]. This legislation places a duty on all NHS trusts, foundation trusts, and Integrated Care Boards to contribute to statutory emissions reduction targets [10]. In practical terms, every NHS trust has been asked to develop a Green Plan to map out a rapid decarbonization trajectory in line with these national goals [11]. These reports cover the organization’s greenhouse gas emissions across Scopes 1 and 2 and a defined subset of Scope 3, aligning with NHS England’s Carbon Footprint (directly controlled emissions) and Carbon Footprint Plus (influenced emissions) frameworks [10]. In short, all NHS trusts are now required to measure and disclose their carbon footprint annually.

No specific mandate exists to isolate AI software emissions in NHS reports, but trusts are expected to account for the carbon impact of digital health technologies under their broader footprint emissions. AI tools are typically subsumed under existing categories such as IT and electricity use. If an AI application runs on hospital-owned servers or devices, its energy consumption contributes to the trust’s Scope 2 electricity emissions, which are calculated using standard conversion factors (eg, kgCO₂ per kWh of UK electricity) [10]. These Scope 2 emissions are reported in line with the Greenhouse Gas Protocol and UK government guidelines [12]. On the other hand, if an AI solution is delivered as a service by a vendor (eg, a cloud-based AI diagnostic tool), the associated emissions fall into Scope 3 (the supply chain or NHS Carbon Footprint Plus). In such cases, trusts rely either on data from the supplier or, more commonly, on estimations since many AI vendors do not disclose product-level carbon data. For AI and other digital services, where direct measurements are rarely available, trusts often resort to input-output modelling or proxy indicators.

Given the data gaps from AI tool suppliers, NHS trusts use established frameworks to estimate emissions from digital technologies in the form of the UK Government’s Greenhouse Gas Conversion Factors, published annually by the Department for Environment, Food & Rural Affairs (DEFRA). This DEFRA framework provides standardized CO₂ equivalent values per unit activity, for example, per kWh of electricity, per passenger-km of travel, or per pound sterling of spend in various economic sectors [13]. This means, if a trust spends a certain amount on IT services or software subscriptions (which would include AI software contracts), that spend can be converted into an estimated carbon emission figure by using the relevant factor. This approach captures the approximate footprint of the wider IT sector per pound spent, rather than the specific energy used by a particular algorithm but is often the only feasible method when suppliers do not provide detailed data. This method had the potential to over, or under, quantity emissions.

There are 3 key problems with this methodology, which contribute to potential underreporting of carbon emissions regarding the use of AI technologies: a lack of granularity, the AI infrastructure life cycle, and unprocured AI tools.

## Problem 1 – Granularity and High-Intensity AI Workloads

Although the above methods allow NHS trusts to include AI in their carbon ledgers, they may not fully capture the true carbon footprint of AI software. One limitation is that of granularity, as generic emission factors provide averages that can obscure the extreme energy intensity of certain AI workloads. For example, one analysis equated training OpenAI’s GPT-3 to the carbon emissions of hundreds of transatlantic flights [14]. Such one-time training emissions would not be visible to a hospital trust procuring the model as a service, since the trust might only log the ongoing usage or subscription cost. Further to this, models are often retrained often to avoid critical issues such as data drift [15], which is unlikely to be evident to those engaged in reporting. Even at the usage stage, AI algorithms can draw significantly more power than conventional software [16]. A recent study warned that implementing large language model systems across hospitals could have very significant environmental consequences, noting that a single complex AI query uses enough electricity to charge a smartphone 11 times (and consumes 20 mL of cooling water), with ChatGPT estimated to use 15 times the energy of a traditional Google search [17]. If a hospital begins running thousands of AI queries on patient data, these energy costs quickly accumulate. Yet, unless the trust actively measures the IT electricity load or the cloud provider reports it, the default accounting might underestimate this. In many cases, the emissions from running hospital AI are simply grouped into facility electricity use, where they are hard to distinguish from other IT or equipment energy demands.

A potential method for overcoming this issue could be to add an AI-specific carbon disclosure clause to digital health contracts. This could, for example, require vendors to provide model-level emissions data (training, retraining, and inference energy use) and data-center carbon intensity by using Greenhouse Gas Protocol methods. Trusts would then record these figures separately rather than folding them into generic IT spend [18,19].

## Problem 2 – AI Infrastructure Life Cycle and Embedded Emissions

Another challenge is that the life cycle impacts of AI infrastructure are often overlooked in current reporting. DEFRA’s conversion factors and NHS models primarily address operational emissions (eg, energy use, fuel, travel). However, AI systems rely on power-hungry hardware (graphics processing units, servers) whose manufacture and maintenance carry a carbon cost that is not accounted for by just looking at electricity consumption. Researchers have highlighted that the embedded carbon in digital hardware is significant, and the manufacturing of high-end processors can roughly double the carbon footprint of AI operations when considered over the hardware’s life [20]. Unless a trust directly purchases new hardware for AI (in which case some of that manufacturing footprint might appear in the supply chain emissions of medical equipment or IT equipment procurement), these upstream impacts remain hidden with current DEFRA estimates. For AI services run in the cloud, the manufacturing footprint is on the vendor’s books and would only reach the NHS if the vendor reports it or if the NHS uses a broad spend-based factor that averages such upstream impacts. In short, today’s estimation techniques might undercount the full lifecycle of emissions from deploying AI.

A consideration for this problem could be to extend Green Plans to include a full life-cycle assessment of AI hardware [21]. This may manifest itself in procurement templates; insist on cradle-to-grave carbon factors for graphics processing units, servers, and cooling equipment; and incorporate them into Scope 3 reporting. This addresses the hidden manufacturing footprint of high-end processors noted in the analysis.

## Problem 3 – Unprocured Generative AI in Daily Practice

The third area where current reporting falls short is on unprocured, freely available, AI such as ChatGPT and Google Gemini. As these are not officially procured by the NHS, and their use by individuals within the health care system is not known or accounted for, their environmental impacts are not reported. The exact usage of these AI tools in the NHS is unknown, but a recent survey of general practice found that 20% of general practices in the United Kingdom use generative AI such as ChatGPT in their everyday practice [22].

Estimates for how much carbon is released per ChatGPT query vary (as OpenAI do not disclose their environmental reports). However, various sources use the figure of 4.32 g of carbon released per prompt [23,24]. In July 2025, 33.6 million general practice appointments took place in the United Kingdom [25]. To estimate the total number per year, we divide this by 30.4 (the average days in a month) and multiply by 365 days in a year to reach 403,421,053 general practice appointments per year. If we assume 20% of these are using unprocured generative AI [22], then we can estimate that each year, 348,555.790 kg of carbon is released from these tools in the general practice alone (see Textbox 1 for estimated calculations).

**Estimated calculations.**

**Estimated number of general practice (GP) appointments per year**

33.6 million GP appointments in July 2025.

(33,600,000 / 30.4) x 365 = 403,421,053 appointments per year

**Estimated total annual unprocured artificial intelligence (AI) queries**

20% use generative AI → 80,684,211 AI queries per year.

**Estimated total annual CO₂ emissions from unprocured generative AI**

Each ChatGPT query emits 4.32 g CO_2_.

80,684,211 × 4.32 g = 348,555,790 g = 348,555.79 kg

Over a year, this is equivalent to around 2,300,000 phlebotomies [26] or 20,000 magnetic resonance imaging scans [27]. Further, a worldwide survey by Elsevier Health of 2607 clinicians [28] found that 48% supported using generative AI to aid clinical decision-making. It is therefore likely that many currently are in the NHS and that the emissions from this are not reported. As the extent of the use of these tools is unknown, there is no way for it to be accounted for in the current reporting mechanisms.

The setting up of a lightweight monitoring for generative-AI web traffic by deploying browser or network logging that counts staff queries to external AI tools and multiplies them by an agreed emission factor may be a way to overcome this lack of reporting. However, this itself raises ethical concerns over privacy [29] and therefore may not be deemed appropriate.

## Moving Forward

Current carbon reporting metrics for the NHS are unlikely to fully capture the environmental impact of AI being implemented. Unlike more traditional sources (energy, travel, waste), which have well-defined reporting protocols, AI as of yet does not have an agreed-upon carbon accounting method in the NHS context. A 2021 scoping review on AI in the NHS noted that standardized measures are lacking for quantifying AI-associated emissions, which limits the ability of health systems to track improvements or trade-offs in this area [3]. More recently, a 2024 policy paper by UK researchers and NHS experts echoed the need for better tools: it called for developing an open and shared database of carbon factors for digital health and for standardizing how we calculate the carbon impact of digital interventions (from electronic records to AI) [30]. The report emphasizes that as health care digitizes, robust preimplementation and postimplementation carbon assessments of new technology are needed to ensure that purported emission savings (eg, through telemedicine or AI-driven efficiency) are not undermined by unaccounted digital emissions.

In practice, NHS trusts currently face data and methodology gaps when it comes to AI. They may know, for example, that a telehealth AI platform reduces patient travel (a clear carbon saving that they can count in reduced transport emissions), but they likely lack precise data on the platform’s own cloud computing footprint. The default approach using DEFRA conversion factors and broad averages is useful but coarse. It treats digital services with a one-size-fits-all lens, which can misrepresent both highly optimized low-carbon software and particularly energy-intensive AI processes. The result is that the carbon footprint of AI in health care is often only partially accounted for. It might be recorded as a small increment in electricity usage or an imputed supply-chain emission, even if the true impact (especially at scale) could be larger.

## Broader Implications

Although this viewpoint focuses on missed AI emissions in NHS reporting, it illustrates a wider challenge in global health care. AI is being implemented without full accounting for externalities, with the environment only one among others such as safety, equity, labor, supply chains, and service resilience. Poorly specified accounting can export carbon and other risks to different regions, weakening international comparisons of AI-enabled care. Progress therefore depends on harmonized disclosure and auditable methods that let health systems judge whether AI genuinely improves care without shifting costs to other people or places.

## Abbreviations

AI: artificial intelligence
DEFRA: Department for Environment, Food & Rural Affairs
NHS: National Health Service
